# Hearing Feelings: Affective Categorization of Music and Speech in Alexithymia, an ERP Study

**DOI:** 10.1371/journal.pone.0019501

**Published:** 2011-05-09

**Authors:** Katharina Sophia Goerlich, Jurriaan Witteman, André Aleman, Sander Martens

**Affiliations:** 1 Neuroimaging Center, University Medical Center Groningen, University of Groningen, Groningen, The Netherlands; 2 Leiden Institute for Brain and Cognition (LIBC), Leiden University, Leiden, The Netherlands; 3 Leiden University Centre for Linguistics (LUCL), Leiden University, Leiden, The Netherlands; University College London, United Kingdom

## Abstract

**Background:**

Alexithymia, a condition characterized by deficits in interpreting and regulating feelings, is a risk factor for a variety of psychiatric conditions. Little is known about how alexithymia influences the processing of emotions in music and speech. Appreciation of such emotional qualities in auditory material is fundamental to human experience and has profound consequences for functioning in daily life. We investigated the neural signature of such emotional processing in alexithymia by means of event-related potentials.

**Methodology:**

Affective music and speech prosody were presented as targets following affectively congruent or incongruent visual word primes in two conditions. In two further conditions, affective music and speech prosody served as primes and visually presented words with affective connotations were presented as targets. Thirty-two participants (16 male) judged the affective valence of the targets. We tested the influence of alexithymia on cross-modal affective priming and on N400 amplitudes, indicative of individual sensitivity to an affective mismatch between words, prosody, and music. Our results indicate that the affective priming effect for prosody targets tended to be reduced with increasing scores on alexithymia, while no behavioral differences were observed for music and word targets. At the electrophysiological level, alexithymia was associated with significantly smaller N400 amplitudes in response to affectively incongruent music and speech targets, but not to incongruent word targets.

**Conclusions:**

Our results suggest a reduced sensitivity for the emotional qualities of speech and music in alexithymia during affective categorization. This deficit becomes evident primarily in situations in which a verbalization of emotional information is required.

## Introduction

Alexithymia (literally translated “no words for feelings”) has been recognized as a major risk factor for a variety of psychopathological and medical conditions, including chronic pain, somatization, depression, and anxiety [1]. This condition is characterized by deficits in the identification and verbalization of one's feelings and the cognitive processing and regulation of emotions [2].

Previous research has shown that alexithymic individuals have difficulty identifying emotional facial expressions [3], [4], matching verbal and non-verbal emotional stimuli [5], and remembering words with emotional connotations [6]. Neuroimaging studies have provided additional evidence for an association of alexithymia with differences in brain activation for a variety of tasks that involve emotional processing, such as the processing of emotional pictures [7] and the processing of facial expressions of emotion [8], the imagery of autobiographical emotional events [9], the observation of fearful body expressions [10], and during empathy for pain [11].

Since such impairment during the conscious processing of emotional information may be dependent upon dysfunctions at earlier processing stages, the investigation of automatic sensitivity to affective stimuli is of great importance to understanding the emotion processing deficits individuals with alexithymia exhibit. Recent studies have suggested impaired processing of emotions even at pre-attentive, automatic processing stages in this condition. When presented with emotionally aversive videos, for instance, individuals scoring high on alexithymia did not show an increase in electrodermal activity as low-scorers on alexithymia did, while no difference in self-reported arousal between high- and low-alexithymics was found [12]. Smaller electrodermal responses were also found in a study using negative masked pictures, likewise suggesting a deficit in early emotional reactivity associated with alexithymia [13]. Other studies, however, report higher autonomic baseline levels in alexithymia [e.g.], [ 14, see 15 for a review]. Aftanas and colleagues measured event-related synchronization (ERS) in participants watching emotional film clips. Results indicated greater emotional reactivity in the right hemisphere in high-scorers on alexithymia, suggesting enhanced negative affect and autonomous arousal associated with this condition [16].

Three recent studies using functional magnetic resonance imaging [17]–[19] further support the view that individuals with alexithymia show impairment during the subconscious processing of emotions. All of these studies assessed the influence of alexithymia on the automatic processing of masked facial expressions of emotions. Sad faces were found to be associated with lower responsiveness of the left [19] and right amygdala [18] as a function of alexithymia. Additionally, Reker and colleagues found reduced activations of the insula, superior temporal gyrus, middle occipital and parahippocampal gyrus in response to sad and happy facial expressions with increasing scores on alexithymia [19]. Masked surprised faces elicited decreased activation of the parahippocampal gyrus and fusiform gyrus as a function of alexithymia [17]. In sum, these studies providence evidence for a hypoactivation of brain areas related to the subconscious processing of facial emotions, suggesting that alexithymia is associated with reduced processing of automatic emotional information.

### Affective Priming

A powerful technique to assess automatic processing of emotions is the affective priming paradigm. The affective priming effect refers to the observation that the affective connotation of a target stimulus, e.g., ‘ugly’ will be judged faster when preceded by an affectively related prime, e.g., ‘hate’ as compared to an affectively unrelated prime, e.g., ‘love’ [20]. The effect is thought to be an early, fast-acting, automatic process that can occur outside of conscious awareness [21], [22]. It has been demonstrated for a variety of stimuli, such as pictures, prosody (“melody of speech”), music, and even odors.

Few studies have employed the affective priming paradigm in alexithymia. The first study to examine affective priming effects as a function of alexithymia was conducted by Suslow [23]. Positive and negative word targets (adjectives) primed by positive or negative words (nouns) were to be pronounced (pronunciation task) or evaluated as positive or negative as quickly as possible (evaluation task). Pearson's correlations revealed no influence of alexithymia on affective word priming during target pronunciation. During affective evaluation, however, alexithymia correlated positively with the affective priming effect for positive word targets, whereas the correlation with negative word targets failed to reach significance [23].

In a follow-up study, the same word evaluation task as in the previous study (word – word prime – target pairs) was employed, and in addition a face evaluation task (face – face prime – target pairs) including happy and sad faces. The positive correlation of alexithymia with affective priming for positive word targets could not be replicated: In both the word evaluation task and the face evaluation task alexithymia did not correlate with the affective priming effect, neither for positive nor for negative targets [24].

In 2002, Suslow and Junghanns [25] employed a lexical decision task on neutral or emotional target words and non-words primed by sentences with congruent or incongruent emotional content. High scorers on alexithymia showed a negative situation priming effect, indicated by faster lexical decisions for targets preceded by affectively incongruent primes.

Vermeulen and colleagues [26] used verbal (positive and negative words) and non-verbal (happy and sad schematic faces) as primes and targets to investigate affective priming effects in alexithymia. Regression analyses on the effect of prime type (happy and angry faces, positive and negative words) showed reduced affective priming with increasing alexithymia scores only for angry face primes, indicative of reduced emotion processing at an automatic level in alexithymia. Based on these findings, the authors suggest a specific impairment during the automatic processing of threatening stimuli (as represented by angry faces) associated with alexithymia [26].

Taken together, previous studies using affective priming paradigms in alexithymia provide preliminary evidence for an impact of alexithymia on the automatic processing of visual emotional stimuli. However, no consistent picture has emerged with respect to the question of whether alexithymia is associated with reduced or increased affective priming effects during the automatic processing of emotions.

### Music and Speech Prosody

An adequate processing of emotional qualities in auditory material such as emotional prosody (“melody of speech”) and music is fundamental to human experience and has profound consequences for functioning in daily life. Both music and speech prosody have been shown to be capable of influencing the processing of visual emotional material [see 27]–[32 for prosody, 28,33–38 for music, see 39 for a review]. To the best of our knowledge, only two previous studies have addressed the impact of alexithymia on the processing of emotions conveyed by speech prosody and music. Swart and colleagues observed no behavioral differences for spoken sentences with incongruent semantics and affective prosody in individuals with alexithymia as compared to controls [4]. Vermeulen and colleagues found that during the presentation of angry, but not happy background music, high scorers on alexithymia recognized fewer anger and joy words than low scorers, indicating hampered memory performance during angry music perception associated with alexithymia [40].

In sum, the literature on the emotion processing deficit in alexithymic individuals demonstrates that alexithymia influences not only the conscious processing of emotion, but that aberrant emotion processing is evident already at very early, automatic processing stages. However, there is no consensus as to whether alexithymia is associated with decreased or increased automatic processing of emotions. In particular, little is known about the manifestation of this automatic processing deficit at the auditory processing level. We investigated the neural signature of automatic affective priming of words, speech prosody, and music as a function of alexithymia by means of event-related potentials (ERPs).

The present study is the first ERP study to investigate the neural signature of auditory emotional processing in alexithymia using affective speech prosody and music in a cross-modal priming paradigm. We hypothesized reduced affective priming effects with increasing scores on alexithymia at the behavioral level. Given the difficulty to identify emotions in alexithymia, we further hypothesized a reduced sensitivity to affective mismatches in alexithymia, reflected in diminished N400 amplitudes in response to affectively incongruent compared to congruent conditions as a function of alexithymia. The results of this study show that alexithymia was indeed associated with diminished N400 amplitudes for affective prosody and music.

## Methods

### Participants

Thirty-two students (16 male, mean age 23.8, SD 4.4) from the University of Groningen participated in the experiment. All participants were right-handed native speakers of Dutch, had normal or corrected-to-normal vision, no hearing impairment and no psychiatric condition in present or past. Participants received €20 for their participation in the two-hour EEG session. The Neuroimaging Center Institutional Review Board approved the experimental protocol and written informed consent was obtained from all participants prior to the study.

### Toronto Alexithymia Scale (TAS-20)

The TAS-20 is the most widely used measure of alexithymia [41], [42] with a demonstrated validity, reliability, and stability [see 43]. A validated Dutch translation of the scale [44] was used for the present study. The scale consists of 20 self-report items rated on a 5-point Likert scale (1: strongly disagree, 5: strongly agree), with five negatively keyed items.

The TAS-20 comprises the subscales: (1) difficulty identifying feelings (e.g., “I often don't know why I'm angry”), (2) difficulty describing feelings (e.g., “I find it hard to describe how I feel about people”), and (3) externally oriented thinking (e.g., “I prefer talking to people about their daily activities rather than their feelings”). Possible scores range from 20 to 100, higher scores indicate higher degrees of alexithymia.

It has been suggested that alexithymia comprises two related, but distinct types [45], [ but see 46], which can be assessed with another self-report questionnaire, the Bermond-Vorst Alexithymia Questionnaire [BVAQ, 45]. Type I alexithymia is thought to be characterized by a general lack of responsiveness to emotion at any level, whereas in type II alexithymia, basic responses to affective stimuli are assumed to be intact, whereas the ability to cognitively access and verbalize them is impaired [45]. Note that the TAS-20 assesses only type II alexithymia. Thus, the findings presented here allow conclusions with regard to type II alexithymia but might not be applicable to type I alexithymia.

Individuals with TAS-20 scores lower or equal to 51 are considered non-alexithymic, a score from 52 to 60 indicates moderate alexithymia. The clinical threshold for alexithymia is a score of 61 [1]. Alexithymia scores of our study sample ranged from 31 to 68 (mean: 43.25, SD: 9.89, median: 41.5, skewness: 0.98).

### Materials

The stimulus set consisted of 48 words for visual presentation (24 positive, 24 negative), 48 pseudo-words spoken in happy (24) and sad (24) prosody, and 48 music segments expressing happy (24) or sad (24) emotion. All stimuli were validated in three separate pilot studies prior to the experiment.

In the visual word pilot, ten independent raters of Leiden University judged the words with emotional connotations on a 9-point Likert scale (−4 = very negative, 0 = neutral, 4 = very positive). Only words rated 3 or higher by 9 out of 10 raters were included as positive word stimuli, only words rated −3 or lower by 9 out of 10 raters were included as negative word stimuli (see table 1).

**Table 1:** List of Affective Words.

	Positive Words	Translation	Negative Words	Translation
1	Bloem	*Flower*	Beul	*Hangman*
2	Bonbon	*Candy*	Bom	*Bomb*
3	Cake	*Cake*	Braaksel	*Vomit*
4	Echtpaar	*Married Couple*	Dief	*Thief*
5	Expert	*Expert*	Galg	*Gallows*
6	Genie	*Genius*	Graf	*Grave*
7	Geschenk	*Present*	Hoer	*Whore*
8	Goedzak	*Good Soul*	Junk	*Junk*
9	Held	*Hero*	Klootzak	*Asshole*
10	Honing	*Honey*	Monster	*Monster*
11	Ijsje	*Ice Cream*	Pijnbank	*Rack*
12	Kanjer	*Hunk*	Pis	*Piss*
13	Lieverd	*Darling*	Pus	*Pus*
14	Maatje	*Buddy*	Racist	*Racist*
15	Paleis	*Palace*	Sadist	*Sadist*
16	Parel	*Perl*	Schijt	*Shit*
17	Ross	*Rose*	Slet	*Slut*
18	Satijn	*Satin*	Sloerie	*Slut*
19	Schatje	*Baby*	Tiran	*Tyrant*
20	Snoep	*Candy*	Tumor	*Tumor*
21	Vriend	*Friend*	Vandaal	*Vandal*
22	Vriendin	*Girlfriend*	Vergif	*Poison*
23	Winnaar	*Winner*	Vetkwab	*Fat Roll*
24	Zon	*Sun*	Viespeuk	*Dirt Bag*

For the prosody pilot, bisyllabic pseudo-words that obeyed Dutch phonotactics were recorded with the help of an actress, cut to a length of approximately 600 ms and amplitude normalized using the Praat speech processing software [47]. The normalization procedure amplified every stimulus item such that the digitalized sample with the maximum amplitude was set at the maximum positive or negative value of the converter range, and all other samples were scaled proportionally. As a result, all stimuli had about equal intensity. Ten independent raters at Leiden University judged the pseudo-words on a 9-point Likert scale (−4 = very sad, 0 = neutral, 4 = very happy). Only pseudo-words rated 3 or higher for happy prosody and −3 or lower for sad prosody by 9 out of 10 raters were included in the study.

Music segments were created from a number of piano pieces by composers of Western classical music (e.g., Bach, Beethoven, Chopin). Segments with a length of 600 ms were excerpted in Praat (cut at zero-crossings), amplitude normalized and judged by 13 independent raters at the University of Groningen on a 9-point Likert scale (−4 = very sad, 0 = neutral, 4 = very happy). Only music segments rated 3 or higher for happy music and −3 or lower for sad music by 11 out of 13 raters were included in the study.

### Procedure

**Figure 1:**
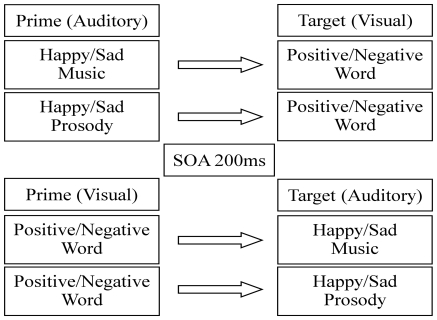
Design of the Cross-Modal Affective Priming Paradigm.

The cross-modal affective priming paradigm included four main conditions (see Figure 1): *MusicTarget* (music targets preceded by visual word prime), *ProsodyTarget* (prosody target preceded by visual word prime), *MusicPrime* (visual word target preceded by music prime), and *ProsodyPrime* (visual word target preceded by prosody prime). Each main condition comprised two congruent and two incongruent sub-conditions (congruent: positive prime – positive target, negative prime, negative target, incongruent: positive prime – negative target, negative prime – positive target).

Each of the four main conditions (*MusicTarget*, *ProsodyTarget*, *MusicPrime*, *ProsodyPrime*) consisted of 96 trials. Overall, each word, prosody and music stimulus was presented twice, once congruent and once incongruent, eliminating stimulus characteristics as an explanation of priming effects. All stimuli (primes as well as targets) were presented for 600 ms. Prime – target pairs were created and presented in a randomized fashion. The four main conditions were presented in four separate blocks, the order of which was presented counterbalanced according to a Latin square.

Stimulus presentation was controlled using E-Prime version 1.2 [48]. Each trial started with a black fixation cross in the middle of the screen (1500 ms), followed by a red fixation cross (500 ms) signaling the occurrence of the prime. When the red fixation cross disappeared, the prime was presented. Two hundred ms after prime onset, the target was presented. An SOA of 200 ms was chosen based on findings that the affective priming effect dissipates after 300 ms [53]. Reaction time was recorded from the onset of the target. To reduce blink artifacts, participants were instructed to blink when the fixation cross was black, and not to blink anymore when it turned red.

The task of the participants was to judge the valence of the word targets (positive or negative) and music and prosody targets (happy or sad) as fast and accurately as possible (affective categorization). Directly after the EEG session, participants completed the TAS-20 questionnaire.

### ERP Recordings

Electroencephalogram (EEG) was recorded from 64 tin electrodes mounted in an elastic electro cap organized according to the international 10/20 system. EEG data were recorded with a linked mastoid physical reference and were re-referenced by using an average reference. Bipolar vertical and horizontal electrooculograms (EOGs) were recorded for artifact rejection purposes.

The ground electrode was applied to the sternum. Impedance of all electrodes was kept below 5 kΩ for each participant. EEG was continuously recorded with a sampling rate of 500 Hz, amplified, and off-line digitally low-pass filtered with a cut-off frequency of 30 Hz. Participants were seated in front of a monitor at a distance of approximately 50 cm in a dimly lit, electrically shielded and sound-attenuated cabin. Music and speech stimuli were presented via loudspeakers placed at the left and right side of the participant at approximately 70 dB.

### Behavioral Data Analysis

**Figure 2:**
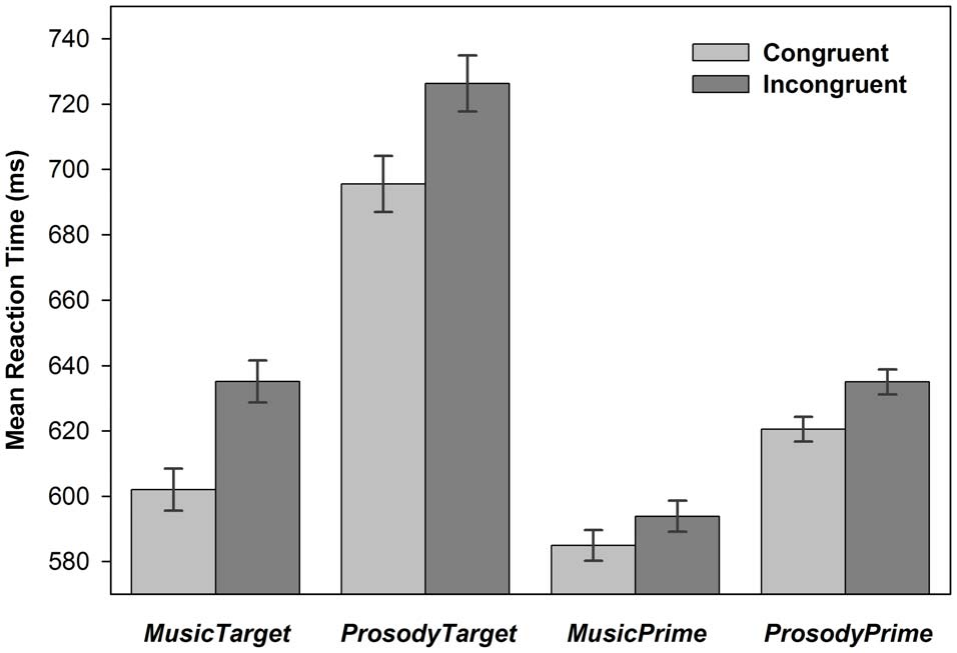
Behavioral Affective Priming Effects. Behavioral affective priming effects during affective categorization of the targets. *MusicTarget*, *p*<0.01, *ProsodyTarget*, *p*<0.01, *MusicPrime*, *p* = 0.07, *ProsodyPrime*, *p*<0.01. Error bars indicate 95% confidence intervals.

First, we aimed to establish the occurrence of cross-modal affective priming in each of the four experimental conditions. To this end, behavioral data were analyzed in a 2 (congruence: congruent vs. incongruent) by 2 (valence: positive vs. negative) repeated-measures analysis of variance (RM-MANOVA) with sex as a between-subjects factor. The analysis of accuracy showed that performance was higher than 90 percent in all conditions, indicating ceiling effects. Therefore, only the results of the reaction time (RT) analyses on correctly identified targets are reported (see Figure 2).

Secondly, the impact of alexithymia on affective priming was tested in a 2 (congruence: congruent vs. incongruent) by 2 (valence: positive vs. negative) repeated-measures analysis of covariance (RM-MANCOVA) with alexithymia as a covariate and sex as a between-subjects factor.

Lastly, Pearson's correlations were conducted to test the impact of alexithymia on differences in reaction time between affectively congruent and incongruent targets. In order to test for an effect of valence, alexithymia scores were further correlated with differences in reaction time for positive and negative targets separately.

### ERP Data Analysis

The EEG data were analysed with Brain Vision Analyzer (version 1.05). Prior to averaging, trials with eye-movement and blink artifacts were excluded from analysis. Criteria for artifact rejection within an epoch were a maximal voltage step of 50 µV, a maximal difference between two values in a segment of 100 µV, and a minimal and maximal amplitude of −100 µV and 100 µV, respectively. A total mean number of 360.1 trials (SD 21.8) was recorded for each of the 32 participants (mean 89.2, SD 5.2 per experimental condition). Artefact rejection excluded a mean percentage of 3.4 percent of all trials (ranging from 0.3 percent to 23.3 percent across participants), leaving a total mean number of 343.7 trials (SD 27.1) for analysis, with a mean number of 85.7 trials (SD 6.7) per experimental condition.

**Figure 3:**
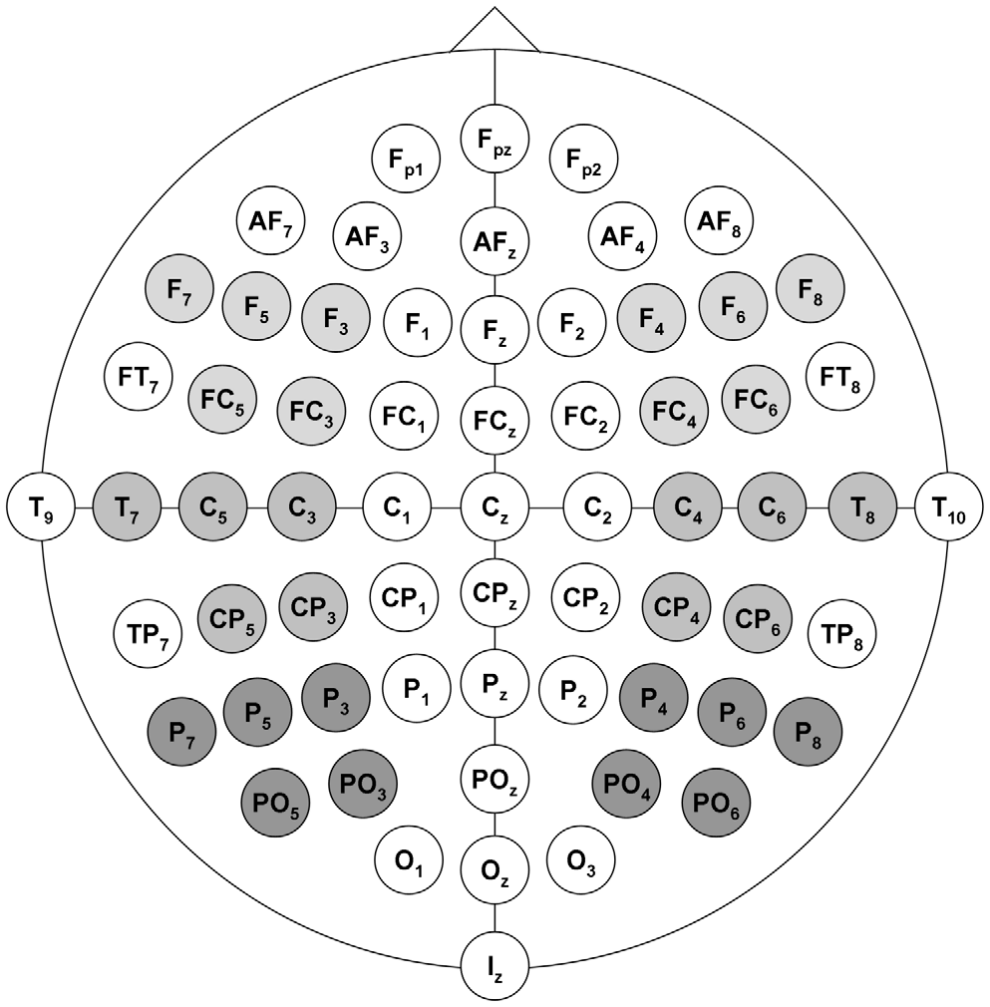
Electrode Map. Map of electrode sites used for analysis with left and right anterior, central, and posterior regions identified.

ERP epochs for each subject were computed in a 1000 ms time-window following the onset of the targets, which were aligned to a 100 ms pre-target baseline. Visual inspection of the data revealed negativities in response to affectively incongruent compared to congruent targets between 400 and 500 ms following the onset of the targets. These negativities were found consistently between 400 and 500 ms for music and prosody targets as well as for visual word targets, indicating that regardless of modality, affectively incongruent targets elicited N400 effects in a time-window of 400–500 ms following target onset. Based on this observation and previous N400 literature, the time-window 400–500 ms post-target onset was chosen for statistical analysis. Mean amplitudes for positive and negative music, speech, and word targets were computed at the N400 time-window (400–500 ms after target-onset) in each participant, beginning at the onset of the targets.RM-MANOVA) was conducted in SPSS (17.0) using a total of 30 electrodes in six topographic regions (anterior, central, posterior) in the left and right hemisphere (see Figure 3). The left anterior region included electrodes F3, F5, F7, FC3, and FC5, the right anterior region electrodes F4, F6, F8, FC4, and FC6. The left central region included electrodes C3, C5, CP3, CP5, and T7, the right central region electrodes C4, C6, CP4, CP6, and T8. The left posterior region included electrodes P3, P5, P7, PO3, and PO7, the right posterior region electrodes P4, P6, P8, PO4, and PO8.To test for an effect of affective congruence (i.e., affective priming) between primes and targets as well as for effects of valence, congruence and valence were entered into the analysis as separate factors. Topographic region and hemisphere were additionally included as within-subject factors. Based on previous findings of sex differences in emotional prosody processing [30]–[32] and the processing of emotions conveyed by music [50]–[52], sex was included as a between-subjects factor. In case of sphericity violations, Greenhouse-Geisser corrected p-values are reported. A Sidak correction of p-values was used in pairwise comparisons between the levels of factors.

Secondly, in order to test for the impact of alexithymia on affective priming and valence of primes and targets, RM-MANCOVA was carried out using the same factors as above and additionally including scores on the alexithymia questionnaire TAS-20 as a covariate.

Lastly, as in previous studies on affective priming in alexithymia [23], [24], [26], correlation analyses were conducted to test the impact of alexithymia on N400 amplitudes in response to an affective mismatch between primes and targets. Given that the N400 reflects the processing and integration of meaning [for a recent review, see 53], its amplitude can be used as an indicator of individual sensitivity to mismatches in affective meaning between stimuli such as music, prosody, and words. To obtain an index of the relative increase in N400 amplitudes in affectively incongruent compared to congruent conditions, N400 mean voltages following congruent target onsets were subtracted from N400 mean voltages following incongruent target onsets at each electrode site.

In order to reduce the number of comparisons and thereby the probability of false positives, correlations were not carried out at the 30 electrode sites separately, but N400 means of the five electrodes contained in each of the six topographic regions (left anterior, central, posterior; right anterior, central, posterior) were collapsed. The resulting N400 amplitude means for the six regions were used in subsequent correlation analyses.

Standardized alexithymia scores (31–68, mean: 43.25, SD: 9.89) were then correlated with the absolute difference in N400 amplitude means between affectively incongruent and congruent conditions. In order to test for effects of valence, Pearson's correlations with alexithymia were further conducted separately for positive and negative targets. For this purpose, separate N400 means for positive and negative targets (indexes of valence effects) were obtained by subtracting N400 means for positive (negative) targets in congruent conditions from N400 means for positive (negative) targets in incongruent conditions.

## Results

### Behavioral Results

#### MusicTarget

RM-MANOVA revealed a significant affective priming effect for music targets primed by words with affective connotations. Participants evaluated music segments preceded by affectively congruent visual word primes significantly faster than music segments preceded by affectively incongruent word primes, as indicated by a main effect of congruence [*F*(1,30) = 27, *p*<0.01]. A main effect of valence [*F*(1,30) = 11.1, *p*<0.05] revealed faster categorization of happy music targets compared to sad music targets (608 ms vs. 630 ms). Further, a main effect of sex was found [*F*(1,30) = 4.2, *p* = 0.05], indicating that female participants categorized affective music targets significantly faster than male participants (590 ms vs. 647 ms).

After controlling for alexithymia in RM-MANCOVA, the effect of congruence [*F*(1,29) = 6.6, *p*<0.05] and sex [*F*(1,29) = 4.2, *p* = 0.05] remained significant. However, the effect of valence failed to reach significance [*F*(1,29) = 2.2, *p* = 0.15]. Alexithymia as a between-subjects effect was not significant [*F*(1,29)<1], and no interactions with alexithymia were observed.

Pearson's correlations revealed no significant impact of alexithymia on the behavioral affective priming effect for music targets preceded by visual word primes (*r* = −.24, *p* = 0.19). There was no effect of valence (*r* = −.15, *p* = 0.42).

#### ProsodyTarget

RM-MANOVA demonstrated a significant affective priming effect for prosody targets primed by words with affective connotations. Prosody targets were evaluated significantly faster when preceded by affectively congruent as opposed to affectively incongruent visual word primes, as indicated by a main effect of congruence for reaction time [*F*(1,30) = 13.1, *p*<0.01]. No main effect of valence was observed [*F*(1,30)<1]. A significant two-way interaction between congruence and valence showed that sad prosody was categorized significantly faster than happy prosody in affectively incongruent, but not congruent conditions (716 ms vs 737 ms, *p*<0.01).

Including alexithymia as a covariate in RM-MANCOVA showed that the effect of congruence remained significant [*F*(1,29) = 6.7, *p*<0.05]. A trend toward an alexithymia×congruence interaction [*F*(1,29) = 3.3, *p* = 0.08] suggested that this affective priming effect tended to be reduced in individuals with higher alexithymia scores. As between-subjects effect, alexithymia was not significant [*F*(1,29)<1]. No main effect of valence was observed [*F*(1,29) = 1.6, *p* = 0.21], and the alexithymia×valence interaction failed to reach significance [*F*(1,29) = 1.3, *p* = 0.27].

Correlation analyses confirmed a trend toward a negative correlation between alexithymia and reaction times for prosody targets preceded by visual word primes (*r* = −.30, *p* = 0.09), suggesting a trend toward reduced affective priming with increasing alexithymia scores. No correlation between alexithymia and the valence of prosodic targets was found (*r* = −.22, *p* = 0.23).

#### MusicPrime

RM-MANOVA showed a trend to categorize affective word targets faster when preceded by affectively congruent vs. incongruent music primes (i.e., affective priming effect) as suggested by a marginally significant effect of congruence [*F*(1,30) = 3.4, *p* = .07]. A main effect of valence was not observed [*F*(1,30)<1]; however, a significant interaction between valence and congruence [*F*(1,30) = 12.9, *p*<0.01] suggested that affective priming by music on words was stronger for positive word targets. The effect of sex was not significant [*F*(1,30)<1].

RM-MANCOVA including alexithymia as a covariate showed no significant main effects or interactions for word targets preceded by music primes.

Correlation analyses confirmed the absence of an effect of alexithymia on affective priming in this condition: no significant correlations were observed between alexithymia and affective congruence (*r* = −.08, *p* = 0.66) and the valence of affective words (*r* = −.23, *p* = 0.21).

#### ProsodyPrime

RM-MANOVA revealed a significant affective priming effect for word targets primed by emotional prosody. Words with emotional connotations were evaluated significantly faster when preceded by affectively congruent as opposed to affectively incongruent prosody primes, as indicated by a main effect of congruence [*F*(1,30) = 14.6, *p*<0.01]. There was no main effect of valence [*F*(1,30)<1] and sex [*F*(1,30) = 1.5, *p* = 0.23]. A significant congruence×valence interaction *F*(1,30) = 12.4, *p*<0.01] showed that the affective priming effect was stronger for positive than for negative words. This effect tended to be qualified by sex differences: a marginally significant three-way congruence×valence×sex interaction [*F*(1,30) = 3.8, *p* = 0.06] suggested that in female participants, affective priming of words was evident regardless of valence, whereas male participants showed affective priming only for positive word targets.

When including alexithymia as a covariate (RM-MANCOVA), the affective priming effect was only marginally significant [congruence: *F*(1,30) = 3.8, *p* = 0.06]. No further main effects were observed in this condition. Alexithymia did not reach significance as between-subjects effect [*F*(1,30)<1] and did not interact with congruence [*F*(1,30) = 1.3, *p* = 0.27] or valence *F*(1,30)<1]. The three-way interaction congruence×valence×sex remained marginally significant [*F*(1,30) = 3.8, *p* = 0.06].

Correlation analyses confirmed the absence of an effect of alexithymia on words primed by emotional prosody: no significant correlations were observed between alexithymia and affective congruence (*r* = −.22, *p* = 0.24) and the valence of affective words (*r* = .04, *p* = 0.84).

### ERP Results

#### MusicTarget

**Figure 4:**
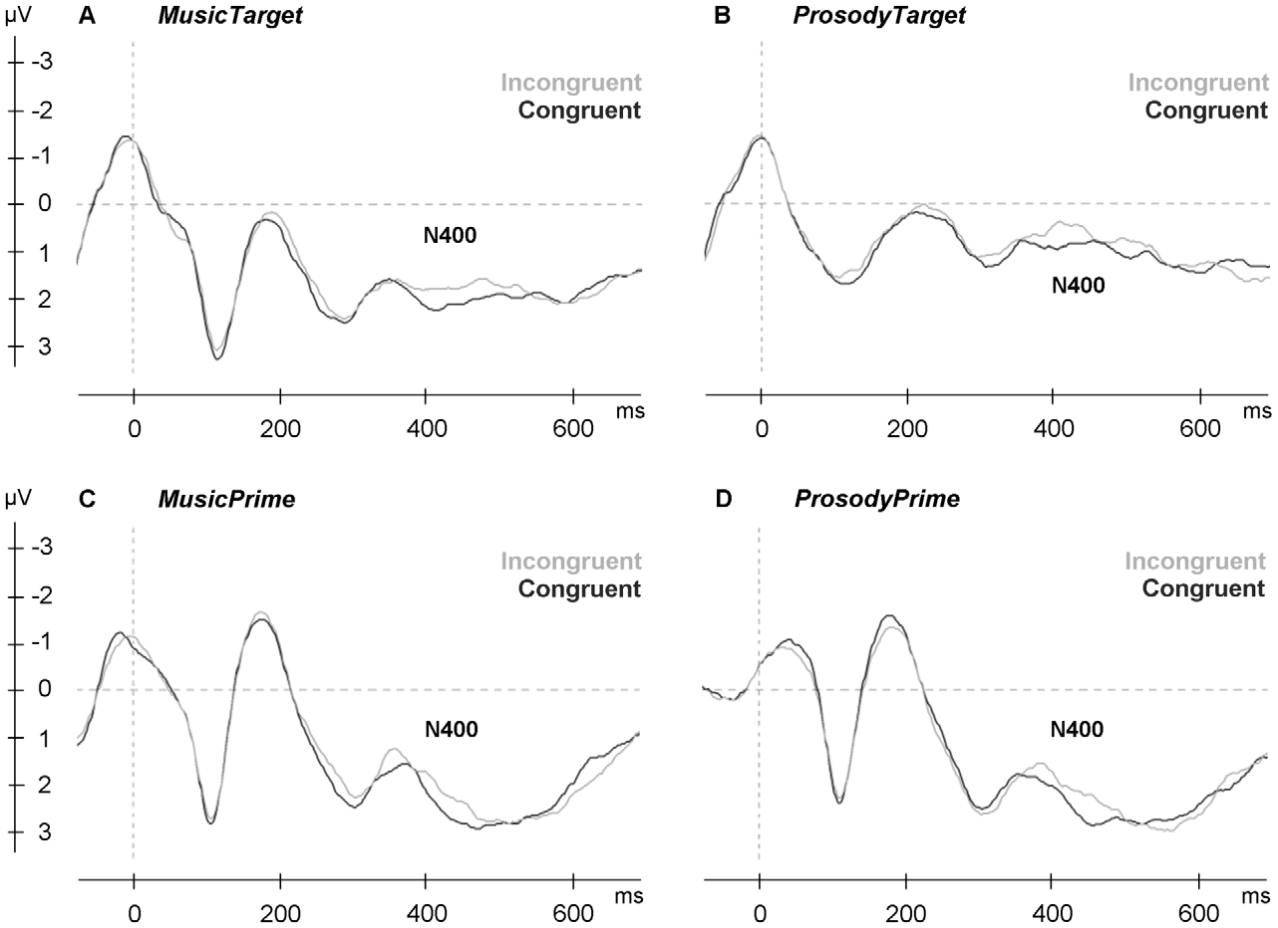
The N400 to Affective Incongruency. N400 in response to affectively incongruent targets (gray) versus affectively congruent targets (black) during affective categorization. Grand averages of 32 subjects at electrode site P3 are shown for A: *MusicTarget*, B: *ProsodyTarget*, C: *MusicPrime*, D: *ProsodyPrime*.

RM-MANOVA revealed a main effect of congruence at the N400 time-window [*F*(1,30) = 4.8, *p* = 0.04], indicating a larger N400 for incongruent compared to congruent music targets (see Figure 4 for all conditions). There was no main effect of valence [*F*(1,30)<1]. A significant congruence×sex interaction [*F*(1,30) = 7.5, *p* = 0.01] indicated that N400 amplitudes were larger in female than in male participants. Further, a significant three-way interaction of congruence×valence×sex [*F*(1,30) = 7.6, *p* = 0.01] suggested that in female participants, affectively incongruent music targets elicited larger N400 amplitudes regardless of valence, whereas in male participants the N400 occurred only for happy music targets. Sex as a between-subject factor did not reach significance [*F*(1,30)<1].

Further, a main effect of region [*F*(1,60) = 96.6, *p*<0.01] showed that negativities were largest at anterior regions. A significant interaction between region and hemisphere [*F*(1,60) = 14.8, *p*<0.01] further suggested more negative voltages at left anterior and central regions (compared to their right counterparts) and more negative voltages at the right posterior region (compared to its left counterpart).

After controlling for alexithymia, RM-MANCOVA yielded no main effect of congruence [*F*(1,29)<1]. However, a significant congruence×sex interaction [*F*(1,29) = 7.4, *p* = 0.01] revealed that the N400 for emotional music occurred only in female participants. The main effect of region remained [*F*(1,58) = 12.1, *p*<0.01]. No further main effects were observed. The two-way interaction region×hemisphere [*F*(1,60) = 14.8, *p*<0.01] remained significant. Alexithymia showed a marginally significant interaction with hemisphere [*F*(1,29) = 4.0, *p* = 0.06], indicating a trend toward larger negativities in the left hemisphere in individuals with higher alexithymia scores.

**Figure 5:**
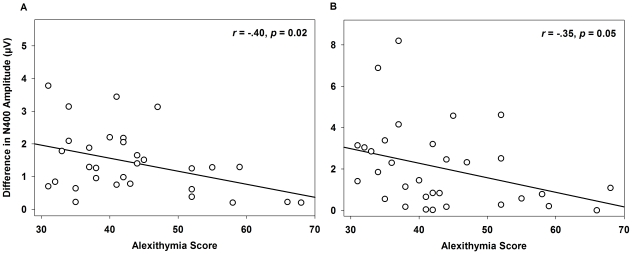
Correlation of Alexithymia with the N400. Panel A: Impact of Alexithymia on the N400 for Happy Music. Negative correlation of alexithymia with amplitudes of the N400 in response to happy music (*MusicTarget*: *r* = −.40, *p* = 0.02) at the left anterior region. Panel B: Impact of Alexithymia on the N400 for Happy Prosody. Negative correlation of alexithymia with amplitudes of the N400 in response to happy prosody (*ProsodyTarget*: *r* = −.35, *p* = 0.05) at the left posterior region.

Pearson's correlations confirmed an association of alexithymia with N400 amplitudes for affectively incongruent compared to congruent music targets (Figure 5). This effect was found to be left-lateralized. For affectively incongruent music irrespective of valence, alexithymia correlated negatively with N400 amplitudes at the left central region (*r* = −.36, *p* = 0.04), and tended to correlate negatively with N400 amplitudes at the left posterior region (*r* = −.33, *p* = 0.07). For happy music targets only, alexithymia also correlated negatively with N400 amplitudes in the left anterior region (*r* = −.40, *p* = 0.02).

#### ProsodyTarget

A main effect of congruence was observed for prosody targets at the N400 time-window [*F*(1,30) = 7.1, *p* = 0.01], indicating a larger N400 for affectively incongruent compared to congruent targets. No main effect of valence was found [*F*(1,30)<1]. A main effect of region [*F*(1,30) = 7.9, *p*<0.01] suggested that most negative voltages occurred at anterior regions. No further main effects or interactions reached significance.

After controlling for alexithymia, the effect of congruence for prosody targets failed to reach significance [*F*(1,29)<1]. RM-MANCOVA revealed no significant main effects. There was a trend toward a congruence×hemisphere interaction [*F*(1,29) = 3.1, *p*<0.09], indicating that the N400 for affectively incongruent prosody tended to be larger in the left hemisphere compared to the right hemisphere. This lateralization effect tended to be stronger in individuals with high scores on alexithymia, as indicated by a marginally significant three-way congruence×hemisphere×alexithymia interaction [*F*(1,29) = 3.5, *p*<0.07].

Pearson's correlations confirmed an association of alexithymia with N400 amplitudes in response to affectively incongruent prosody (see Figure 5). This effect was found to be located at the posterior region in the left hemisphere. Alexithmia showed a significant negative correlation with N400 amplitudes in response to happy prosody (*r* = −.35, *p* = 0.05), and tended to correlate negatively with N400 amplitudes to sad prosody (*r* = −.30, *p* = 0.09).

#### MusicPrime

For words primed by affective music, RM-MANOVA revealed no significant effect of congruence [*F*(1,30)<1] or of valence [*F*(1,30)<1]. No significant interactions with congruence were found. Thus, the occurrence of an N400 effect to affectively incongruent words primed by music could not be established.

The effect of alexithymia when included as a covariate was not significant [*F*(1,29)<1]. Congruence [*F*(1,29) = 1.8, *p* = 0.18] and valence [*F*(1,29)<1] remained insignificant.

As RM-MANOVA and RM-MANCOVA showed that there was no N400 in response to affectively incongruent words primed by music, Pearson's correlations between alexithymia and N400 amplitudes could not be conducted in this condition.

#### ProsodyPrime

A main effect of congruence was observed for visual word targets preceded by prosody primes at the N400 time-window [*F*(1,30) = 6.6, *p* = 0.02], indicating a larger N400 for affectively incongruent compared to congruent target words. No main effect of valence was found [*F*(1,30)<1]. The congruence effect was qualified by a three-way congruence×valence×hemisphere interaction [*F*(1,30) = 4.6, *p* = 0.04], which indicated that in the left hemisphere, the N400 was elicited by both positive and negative words, while in the right hemisphere this was only true for negative words.

A main effect of region [*F*(1,60) = 50.7, *p*<0.01] suggested more negative voltages at anterior (mean: −1.9 µV) and central regions (mean: −1.0 µV) compared to posterior regions (mean: 2.5 µV). The effect of sex reached significance [*F*(1,30) = 4.8, *p* = 0.04], indicating more negative voltages in females as compared to male participants. A significant region×hemisphere interaction [*F*(1,60) = 14.3, *p*<0.01] showed more negative voltages in the left anterior region, but more negative voltages at central and posterior regions in the right hemisphere. This interaction was further qualified by a three-way interaction region×hemisphere×sex [*F*(1,60) = 6.7, *p*<0.01], suggesting more negative voltages at right central and posterior regions in both genders, and more negative voltages in the left anterior region in females but no anterior lateralization in male participants.

Including alexithymia as a covariate in RM-MANCOVA, the congruence effect became insignificant [*F*(1,29)<1], instead a main effect of valence was observed [*F*(1,29) = 4.5, *p* = 0.04], indicative of more negative voltages in response to negative compared to positive target words. Alexithymia interacted with this valence effect [*F*(1,29) = 4.7, *p* = 0.04]: for negative targets the difference in voltages did not vary as a function of alexithymia, whereas positive words elicited more negative voltages with increasing alexithymia scores. However, the interaction congruence×valence×alexithymia was insignificant [*F*(1,29)<1], indicating that the interaction between alexithymia and valence did not qualify the N400 effect to incongruent vs congruent conditions. As a between-subjects effect, alexithymia was not significant [*F*(1,29)<1]. The effect of region remained significant [*F*(1,58) = 8.4, *p*<0.01], and so did the effect of sex [*F*(1,29) = 4.4, *p* = 0.04]. Lastly, the three-way interaction region×hemisphere×sex was still significant [*F*(1,58) = 6.3, *p*<0.01].

Pearson's correlations confirmed that there was no significant impact of alexithymia on N400 amplitudes in response to affective words primed by prosody (*p*>0.1). No effect of alexithymia on the valence of the word targets was observed (*p*>0.1).

## Discussion

The results of the present study indicate that alexithymia is associated with impairment in the automatic processing of emotion conveyed by music and speech prosody during affective categorization. At the electrophysiological level, alexithymia scores correlated negatively with amplitudes of the N400, an ERP component indicative of individual sensitivity to affective incongruence. This correlation was observed in the left hemisphere in response to affectively incongruent music and speech prosody. For prosodic targets, the effect had a posterior locus; for music targets, differences in N400 amplitudes were more broadly distributed over central and posterior regions for happy and sad music, and additionally included the left anterior region for happy music targets only. No difference was found during affective categorization of word targets. At the behavioral level, we observed a trend toward a reduced affective priming effect with increasing alexithymia scores for prosodic targets (irrespective of valence), and no impact of alexithymia on affective priming for music and word targets.

The results of the present study replicate previous findings of cross-modal affective priming effects between speech prosody and visually presented words [27]–[32] and between music and linguistic stimuli [28], [33]–[39]. Our results further confirm the occurrence of an N400 effect in response to affectively incongruent music, prosody, and linguistic stimuli in a cross-modal priming paradigm [34], [37], [38]. However, we failed to replicate the N400 effect for words primed by affective music. In contrast to the studies by Steinbeis and colleagues [37], [38], we used short natural music excerpts rated as happy or sad instead of music chords. Dissonant chords such as used in those previous studies are perceived as unpleasant, which was presumably (though not formally tested) not the case for our sad piano music excerpts. This difference in pleasantness of the stimuli used could account for the higher potency of chords in priming visual words compared to natural music excerpts such as used in the present study. Our finding of an only marginally significant affective priming effect for music primes at the behavioral level supports this hypothesis.

Furthermore, our findings support the notion of sex differences in the perception of emotion in music and prosody. Female participants categorized both happy and sad music targets faster than men in the present study. This behavioral difference was accompanied by significantly larger N400 amplitudes in response to affectively incongruent music in women compared to men. In addition, women showed an N400 effect irrespective of music valence, whereas in men this effect was only observed for happy music. These differences, indicative of a higher sensitivity to musical emotions in women are in line with previous reports of larger brain activation and greater positive attribution to affective music in women compared to men [50], larger networks of coherent brain oscillations in response to pleasant music in female participants [51], and greater psychophysiological reactivity reflected by elevated finger temperature and skin conductance level in women compared to men [52].

Sex differences in emotional prosody perception have likewise been reported repeatedly: women recognized emotional prosody faster than men [31], and showed an N400 in an emotional prosody Stroop task while no such effect was observed in men [30]. Even at pre-attentive processing levels sex differences seem to exist: deviants in emotional prosody elicited larger amplitudes of the Mismatch Negativity (MMN) in response to prosodic deviants in women, but not in men [32]. We did not find significant effects of sex for emotional prosody targets, however, when prosody served as a prime women tended to show an affective priming effect for word targets irrespective of target valence, whereas this effect occurred in men only for positive word targets. This behavioral difference was accompanied by generally larger negativities to words primed by emotional prosody in women compared to men (an effect not found when the same words were primed by emotional music). The absence of a sex difference for prosody as a target in our study could be due to the low task difficulty, indicated by a ceiling effect in performance. Low task difficulty may have masked possible differences in emotional prosody categorization in our study, and could explain why sex differences did occur when emotional prosody functioned as a prime, but not as a target.

The present study is the first to provide electrophysiological evidence for an emotional categorization deficit in alexithymia for music and speech prosody. Our finding of decreased N400 amplitudes both for emotions conveyed by music and by speech prosody seems conceivable given that music and speech prosody have been shown to use the same acoustic features to convey emotions [54]–[56]. In light of this similarity, it seems reasonable to assume that individuals with alexithymia, a condition characterized by a difficulty to identify emotions, will exhibit comparable differences in brain responses to emotions conveyed by both music and speech prosody. Future studies should employ auditory affective material to provide further evidence for the generality of the emotion processing deficit in alexithymia, which so far has been investigated using almost exclusively visual emotional information.

Our results of reduced N400 amplitudes during affective priming in response to emotional music and speech as a function of alexithymia confirm and extend previous findings of studies employing electroencephalography (EEG) and functional magnetic resonance imaging (fMRI) that have indicated impaired subconscious processing of visual affective material in alexithymia. Corresponding to our finding of reduced left-hemispheric N400 amplitudes for affective music and speech, decreased early theta synchronization (brain oscillations related to the cortico-hippocampal-limbic interaction during cognitive-emotional processing) was observed in the left hemisphere of alexithymic individuals during the processing of emotional pictures. This was interpreted as a disruption in automatic affective processing and as an analytical, categorical decoding difficulty of emotional stimuli in alexithymia [16]. Our findings are further in line with fMRI studies on the automatic processing of facial expressions of emotions in alexithymia. These studies found that alexithymia was associated with reduced activation of several brain areas during the automatic processing of masked happy, sad, and surprised faces [17]–[19], suggesting that alexithymia is associated with reduced emotional processing at an automatic processing level.

The observed trend toward a negative correlation between alexithymia and affective categorization of prosodic targets at the behavioral level is partially in line with a previous study [4]. Although individuals with alexithymia showed lower accuracy and longer reaction times during prosody identification of sentences with mismatching prosody and semantics in that previous study, these differences did not reach significance. Possibly, this was due to the long duration of the sentences presented (20 s), while the current study employed very short prosodic targets with a length of 600 ms, thereby increasing task demands.

We did not find a relationship between alexithymia levels and N400 amplitudes during affective categorization of visual word targets following music and prosody primes. This finding reveals an asymmetry regarding the effect of alexithymia on the processing of emotional music and prosody: During the categorization of word targets primed by emotional music and prosody, alexithymia had no significant impact on N400 amplitudes. In contrast, when affective music and prosody targets were to be categorized, alexithymia scores were associated with significantly decreased N400 amplitudes. This asymmetry for affective categorization of music and prosody targets versus word targets could be due to the fact that in order to categorize the auditory targets (music and prosody), they had to be (internally) verbalized before they could be categorized as happy or sad. In contrast, in conditions in which music and prosody served as primes, such verbalization of auditory affective information was not necessary as the decision was to be made on the visual word targets.

Difficulties to identify and verbalize emotions are diagnostic criteria of alexithymia [57]. Hitherto, the question as to whether alexithymia is associated with impairment in verbalizing emotions conveyed by prosody and music has not been addressed. However, alexithymia exhibits a high comorbidity with Autism Spectrum Disorder (ASD) [58], [59]. In a recent study on the experience of music in ASD, Allen and colleagues report that individuals with diagnoses on the autism spectrum showed conscious awareness of the emotional arousal induced by music, but exhibited limitations in the terms used to describe the emotional effect of music [60]. Such difficulty to verbalize emotions conveyed by music could underlie our observation of diminished N400 amplitudes with increasing alexithymia levels during the affective categorization of emotional music and prosody targets, but not word targets.

Our finding of unaffected emotional word processing is seemingly at odds with previous studies suggesting an impact of alexithymia on affective priming for word targets [23], [26]. However, it should be born in mind that the larger affective priming effect for positive word targets with increasing scores on alexithymia [23] could not be replicated in a follow-up study [24], despite the fact that the same word evaluation task was employed in a larger sample of participants. Instead, the follow-up study revealed no correlations between alexithymia and the affective priming effect, neither for positive word targets nor for negative word targets. Our findings confirm these results. Moreover, Suslow and colleagues used an SOA of 300 ms, although it has been shown that strategic components can come into effect below an SOA of 300 ms [61] and that the affective priming effect dissipates already at 300 ms [49]. Therefore, an involvement of non-automatic, strategic processes cannot be ruled out in these studies.

Vermeulen and colleagues showed that alexithymia correlated negatively with the affective priming effect for word targets when these were primed by angry faces [26]. This reduced affective priming effect was not found for happy face primes and neither for positive and negative word primes. The authors interpreted this finding as an anger/threat-related automatic processing deficit associated with alexithymia. This interpretation found further support in findings of hampered memory performance during the perception of angry, but not happy background music in alexithymia [40]. The present study used happy and sad prosody and music as primes for affective word targets and found that alexithymia did not correlate with the affective priming effect for word targets. These results do not contradict the findings of Vermeulen and colleagues [26], nor can they confirm these results as angry emotion was not included in our paradigm. It would be interesting to test in future studies whether the hypothesis of an anger-specific processing deficit in alexithymia holds when angry speech prosody and anger/threat evoking music are used to prime word targets during affective categorization.

### Limitations

It should be kept in mind that the alexithymia construct may comprise two related, but distinct types, type I and type II alexithymia [45], though a recent study failed to find empirical support for this distinction [46]. The TAS-20 questionnaire, used here in agreement with previous studies on affective priming in alexithymia [23]–[26], [40], covers only type II alexithymia. This type is characterized by deficits to cognitively access and verbalize emotions, while the general emotional responsiveness is thought to be intact. Type I alexithymia, characterized by a general lack of emotional responsiveness could not be controlled for in the present study. Varying scores on type I alexithymia might have confounded the present results and could contribute to the fact that the present findings are not in line with previous studies on affective priming in alexithymia, which likewise used the TAS-20 and thus did not control for this possible confound. Future studies should additionally use the BVAQ questionnaire, which distinguishes between the two types of alexithymia and would thus makes it possible to control for this possible confound.

A further limitation of the present study is the lack of a correction for multiple comparisons during product-moment correlations between alexithymia and N400 amplitudes in response to affective words, music, and prosody. The number of comparisons conducted in the present study was relatively small, however, the present findings should be treated as preliminary for this reason. Future studies should attempt to overcome these limitations.

### Conclusions

In sum, the results of this study suggest a reduced sensitivity to emotional qualities of speech and music in alexithymia at a neurophysiological level. Our findings of differential brain responses to affective categorization of music and speech prosody as compared to visual words with emotional connotations indicates that alexithymia impairs the categorization of affective stimuli primarily in situations in which a verbalization of the emotional information is required. However, this interpretation remains speculative until future research provides further insight into the nature of the emotional processing deficit in alexithymia.
